# High-Throughput Chromatographic Separation of Oligonucleotides: A Proof of Concept Using Ultra-Short Columns

**DOI:** 10.1021/acs.analchem.3c01934

**Published:** 2023-06-29

**Authors:** Honorine Lardeux, Szabolcs Fekete, Matthew Lauber, Valentina D’Atri, Davy Guillarme

**Affiliations:** † Institute of Pharmaceutical Sciences of Western Switzerland (ISPSO), University of Geneva, Geneva 1211, Switzerland; ‡ School of Pharmaceutical Sciences, University of Geneva, Geneva 1211, Switzerland; § Waters Corporation, located in CMU-Rue Michel Servet 1, Geneva 1211, Switzerland; ∥ Waters Corporation, Milford, Massachusetts 01757, United States

## Abstract

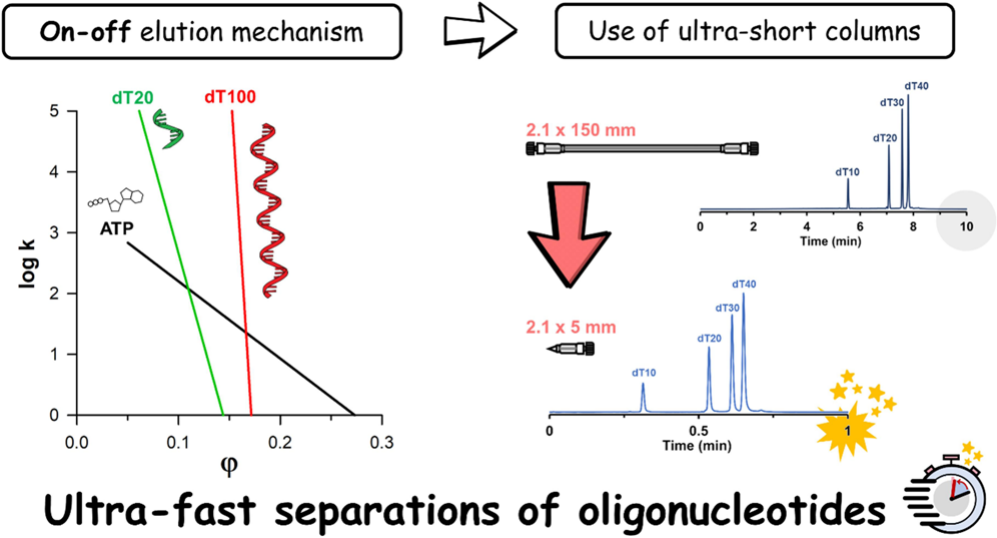

Ion-pairing reversed-phase liquid chromatography (IP-RPLC) is the reference separation technique for characterizing oligonucleotides (ONs) and their related impurities. The aim of this study was to better understand the retention mechanism of ONs, evaluate the applicability of the linear solvent strength (LSS) retention model, and explore the potential of ultra-short columns having a length of only 5 mm for the separation of model ONs. First, the validity of the LSS model was evaluated for ONs having sizes comprised between 3 and 30 kDa, and the accuracy of retention time predictions was assessed. It was found that ONs in IP-RPLC conditions follow an “on–off” elution behavior, despite a molecular weight lower than that of proteins. For most linear gradient separation conditions, a column length between 5 and 35 mm was found to be appropriate. Ultra-short columns of only 5 mm were therefore explored to speed up separations by considering the impact of the instrumentation on the efficiency. Interestingly, the impacts of injection volume and post-column connection tubing on peak capacity were found to be negligible. Finally, it was demonstrated that longer columns would not improve selectivity or separation efficiency, but baseline separation of three model ONs mixtures was enabled in as little as 30 s on the 5 mm column. This proof-of-concept work paves the way for future investigations using more complex therapeutic ONs and their related impurities.

Antisense oligonucleotides (ASOs) are short, synthetic single-stranded oligonucleotides altering RNA processing and hence influencing protein expression. They belong to an emerging class of therapeutic agents toward infectious agents and degenerative diseases. Their success is defined by their ability to target any gene of interest and thereby modulate its expression through Watson–Crick base pairing.^1^ By expanding the range of druggable targets beyond what can be achieved with conventional drugs (*i.e.*, small molecules and antibody-based products),^2^ ASOs and oligonucleotides could offer treatment perspectives for a wide range of previously untreatable diseases. To date, 16 oligonucleotide therapeutics, including 8 antisense therapies, have been approved by the Food and Drug Administration (FDA).^3^

These highly complex drug modalities, whose average size is typically between 15 and 25 nucleotides, are challenging to analyze. Ion-pairing reversed-phase liquid chromatography (IP-RPLC) has become a reference separation technique for characterizing the active principle ingredient (API) product and its related impurities. It is generally accepted that the separation in IP-RPLC is driven by the combination of two retention mechanisms: (i) ion pairing occurs within the mobile phase, followed by binding of the ion pair to the stationary phase (SP) and (ii) ion-pairing agent binds first to the SP and then the ion-pairing process occurs at the surface of the SP.^4,5^

Multiple retention models have been proposed for retention prediction and separation optimization in RPLC.^6^ These include semi-empirical models that aim to describe the relationship between the retention factor (k) and the solvent composition (φ). The linear solvent strength (LSS) model assumes a linear relationship between the logarithm of k and φ.^7^

It is the simplest and most widely used retention model, among other semi-empirical models like Snyder–Soczewiński, Neue–Kuss, Schoenmakers, Slab, and other mixed or polynomial models.^8−10^

Most studies have shown that the RPLC retention can be approximated by the LSS model for many applications, from small molecule to large protein separations.^11^ For large proteins, very often a particular elution mechanism is observed. Their retention is very sensitive to the mobile phase composition, with the protein being infinitely bound at the column inlet until a small change in mobile phase strength allows its complete elution without further interaction.^12,13^ Column length has therefore little impact in controlling their retention, and shorter columns could be considered.^14^ Known as the “bind-and-elute” or “on–off” elution mechanism, it contrasts with the multistep partitioning process that explains the elution of small molecules.

Several retention time prediction models for IP-RPLC analysis of oligonucleotides have been developed over the last 20 years, mostly using modeling and machine-learning-based approaches.^15−19^ Few studies have reported the use of the LSS model for oligonucleotide separations. Liang et al.^20^ demonstrated that this linear model is applicable to describe the isocratic retention of 5- to 30-mer oligonucleotides and that the retention is highly sensitive to changes in mobile phase composition. This linear model has also been assumed to be valid for oligonucleotides in recent works by Fekete and Lauber^21^ as well as Fornstedt and Enmark.^22^

The aim of this work was to study the applicability of the LSS model to oligonucleotides’ IP-RPLC separations in gradient elution mode and to explore the potential of ultra-short columns (*i.e.*, 5 mm in length) to speed up separations. In this proof-of-concept study, a homologous series of oligodeoxythymidines (dT10 to dT100, ranging from 3 to 30 kDa) was considered to study the retention behavior of oligonucleotides. First, the validity of the LSS model was assumed, and the LSS parameters were evaluated. The accuracy of the retention time predictions was then assessed to verify the appropriateness of the linear model, and the effective column length (*L*_eff_) was also estimated. After considering the impact of the instrumentation on the efficiency, and in particular the injected volume and the geometry of the post-column tube, various column lengths of 150, 50, and 5 mm were systematically compared. Finally, optimized ultra-fast separations (30 s) were developed on the shortest column for three oligonucleotide mixtures.

## Experimental Section

### Chemicals and Samples

Water was obtained from a Milli-Q water purification system from Millipore (Bedford, MA). LC-MS grade methanol (MeOH) was purchased from Thermo Fisher Scientific (Reinach, Switzerland). Oligonucleotides were purchased from Eurogentec (Seraing, Switzerland) and Integrated DNA Technologies (IDT, Leuven, Belgium). 1,1,1,3,3,3-Hexafluoro-2-propanol (HFIP, ≥99%), triethylamine (TEA, ≥99.5%), and RNase-free water were purchased from Sigma-Aldrich (Buchs, Switzerland).

### Sample and Mobile Phase Preparation

100 μM oligonucleotide aliquots were initially prepared by reconstituting lyophilized material in the appropriate volume of RNase-free water and stored at −20 °C.

Equimolar oligonucleotide mixtures were prepared by mixing aliquots and diluting the products to 5 μM in RNase-free water prior to the IP-RPLC analysis (if not stated otherwise). Sample mixtures used to illustrate the retention of oligonucleotides were (first part of the study) the following: dT10–40 (a mixture of seven poly-deoxythymidylic (dT) acids of 10-, 15-, 20-, 25-, 30-, 35-, and 40-mer) and dT40–100 (a mixture of four poly-dT acids of 40-, 60-, 80-, and 100-mer). For the second part of the study, dT40–100, 4 dT (a mixture of four poly-dT acids of 10-, 20-, 30-, and 40-mer), and 4 PS (a mixture of four 20-mer poly-dT acids with 0, 6, 9, and 19 phosphorothioate modifications that replace the phosphodiester linkages) were used.

Mobile phase A was 14 mM TEA in water, containing 100, 160, or 400 mM HFIP, pH 8.2, 8.1, or 7.8, respectively. Mobile phase B was a mixture of 50:50 mobile phase A and methanol.

### Chromatographic System, Columns, and Software

All chromatographic separations were performed on a Waters ACQUITY UPLC I-Class System (Milford, MA) equipped with a binary solvent delivery pump, an autosampler with a flow-through-needle (FTN), and a UV detector with a 0.5 μL UV flow cell. The overall extra-column volume was measured as 5.6 μL from the injection seat of the autosampler to the detector cell, while the offset time was equal to 0.87 s. These values were found after plotting system residence time *vs* reciprocal flow rate (1/F) for various experiments conducted at several flow rates in the absence of a column (zero-dead volume union). Waters ACQUITY UPLC BEH C18 1.7 μm Column, 5 mm × 2.1 mm, 130 Å VanGuard Pre-column was used in this study. Commercial 50 mm and 150 mm × 2.1 mm columns (Waters ACQUITY Premier Oligonucleotide BEH C18 1.7 μm, 130 Å Column) were also used. Data acquisition and instrument control were performed by Empower 3 Software (Waters). The freely available Excel Spreadsheet developed by Guillarme *et al.* was used to derive LSS parameters and predict retention times.^23,24^

### Apparatus and Methodology

Sample volumes of 1 μL were injected using linear gradients (for all separations). The column temperature was set at 60 °C unless stated otherwise. Various semi-empirical models are used in LC to describe the relationship between experimentally observed retention times and mobile phase composition or gradient conditions. In this study, the following model was considered
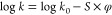
1where *k* is the retention factor, φ is the volume fraction of the organic mobile phase (stronger eluent), *S* is a constant for a given solute at fixed experimental conditions, and *k*_0_ is the extrapolated value of *k* for φ = 0.

The relative solute migration velocity (*u*_rel_) then can be expressed as
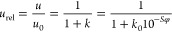
2where *u* is the solute’s migration velocity and *u*_0_ is the interstitial mobile phase velocity.

To evaluate the retention behavior of oligonucleotides in IP-RPLC and verify the applicability of the LSS model, dT10–40 and dT40–100 mixtures were analyzed.

To derive the log *k*_0_ and *S* parameters, initial gradients of 10 and 30 min were first performed. Detailed conditions and experimentally obtained LSS parameters are reported in Tables S1 and S2, respectively.

To study the adequacy of the LSS model and the accuracy of retention time prediction (extrapolation and interpolation), gradients of 5, 15, 20, and 60 min were run using the same conditions. Then, experimentally observed retention times (*t*_r,exp_) were compared with the predicted retention times (*t*_r,pred_). The corresponding errors (%) are reported in Table S3 and calculated as follows
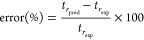
3

To visualize the retention behavior in RPLC of an intact monoclonal antibody (mAb)^14^*vs* IP-RPLC of ATP, 20- and 100-mer oligonucleotides, plots of log *k* = *f*(φ) and *u*_rel_ = *f*(φ) were constructed.

Effective column lengths resulting in a predefined exit retention factor (e.g., *k*_e_ = 0.5) were calculated for a range of flow rates and gradient times, assuming a Δ*B* range of 20%, based on a recently proposed procedure.^21^

For the second part of the study, the mobile phase resulting in the highest *S* values (containing 100 mM HFIP in MPA) was selected for the separation of the 4 dT mixture (dT10, dT20, dT30, dT40), which had been previously concentrated to 15 μM to increase sensitivity.

Peak capacity (*P*), sometimes referred to as average peak capacity, was calculated using the average peak width at half-height (*w*_50%_) and considering the elution window between the last and first peaks (*t_n_* – *t*_1_) and determined according to the following equation
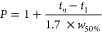
4

The minimum peak capacity (*P*_min_) was calculated for the critical pair of a mixture using the average peak width at half-height of the peaks (*w*_50%(1-2)_) and their difference in retention times (*t*_2_ – *t*_1_) and determined according to the following equation^25^
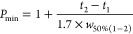
5

To evaluate the impact of instrumentation, the injection volume and the column outlet tubing were systematically varied. All chromatographic conditions for the instrumentation study are provided in Table S4. To compare columns of different lengths, gradients of 10–50%B were performed using various flow rates (*i.e.*, 0.25, 0.50, and 1.00 mL/min) and gradient times (*i.e.*, 1, 10, and 30 min).

Finally, reference separations were obtained in 1 min on a 5 mm column at a flow rate of 1 mL/min. For 4 dT, dT40–100, and 4 PS, gradients were 10–50%B, 28–40%B, and 20–40%B, respectively. High-throughput separations were then performed at 90 °C, at a high flow rate (*F* = 1.75 mL/min) and by running a fast gradient (*t*_G_ = 30 s). For 4 dT, dT40–100, and 4 PS, the corresponding gradients were 10–26%B, 21–27%B, and 16–24%B, respectively.

## Results and Discussion

### Retention Properties of Oligonucleotides

Several studies have reported the elution behavior of ONs in IP-RPLC in the presence of various ion-pairing reagents and using different stationary phases.^25−27^ Efforts were mainly focused on (1) improving the chromatographic resolution (selectivity and peak capacity) of ONs or (2) improving the LC-MS sensitivity.^25^ Relative retention indices and retention time calibration of homo-oligonucleotides in IP-RPLC have been published,^28^ but absolute dimensionless measures of retention (like the S parameter of the LSS model) have not been reported yet, at least to the best of our knowledge. In this study, we focus our attention especially on the sensitivity of solute retention to mobile phase composition (*S* parameter), as this determines the required (effective) column length. We also discuss the log *k*_0_ parameter that corresponds to the extrapolated value of *k* for φ = 0. Here, the change in mobile phase composition was approximated by the change of organic modifier composition (methanol), while neglecting the effect of the small change in additive concentration.^29^ It has also been reported that IP-RPLC separations of ONs benefit from shallow gradients, as they elute in relatively sharp peaks, despite the shallow gradient applied.^25^ This may indicate that the retention of the solute is very sensitive to the eluent composition (on–off-like behavior). In practice, 50–150 mm long columns are often used for the IP-RPLC analysis of ONs, which is not necessary if the on–off behavior is valid.

First, a simple data treatment based on linear regression (eq 1) was applied to determine the log *k*_0_ and *S* parameters of various homo-oligonucleotides (10- to 100-mer).^24^ The methodology and chromatographic conditions are described in the section Apparatus and Methodology and Table S1, respectively. *S* values in the range of 23–263 were obtained, with log *k*_0_ values varying from 3.5 to 48 (Table S2). The model parameters were also determined for a small molecule, namely, adenosine triphosphate (MW = 507 Da, log *k*_0_ = 3.5, *S* = 12.7).

**Figure 1:**
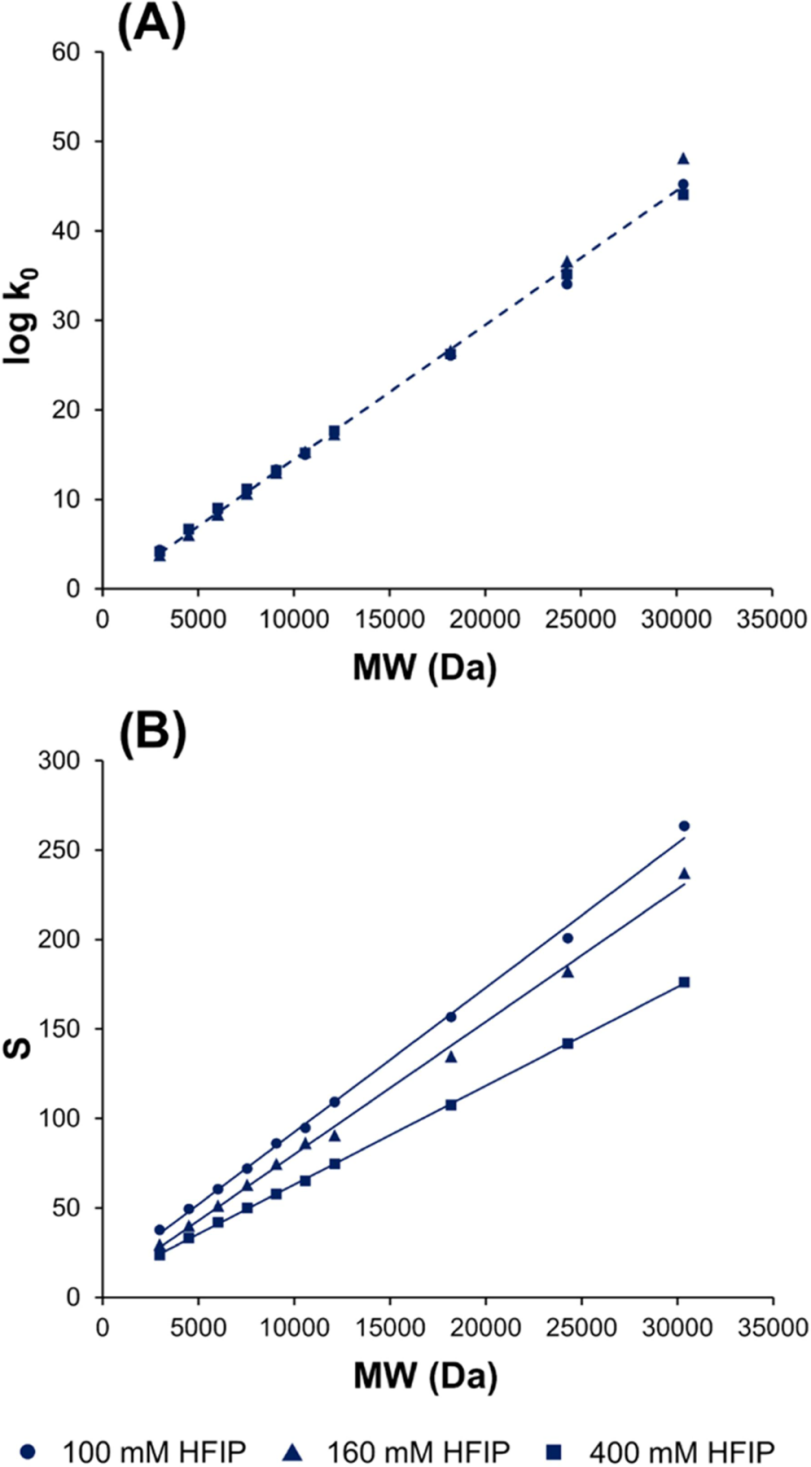
Experimentally observed log *k*_0_ (A) and *S* (B) values as a function of molecular weight (MW) for poly-deoxythymidine oligonucleotides in IP-RPLC mode. Values obtained at fixed experimental conditions with mobile phase A containing 100, 160, or 400 mM of HFIP.

The derived model parameters were used to predict retention times for various gradient conditions. The accuracy of the predictions was estimated by calculating the errors (%) between the predicted and experimental retention times, as reported in Table S3. The error on retention time prediction was very low for both interpolated and extrapolated gradient time ranges. The error was typically <1% (only one ON showed a prediction error of 1–2.5%). This confirms the applicability of the LSS model to the 10- to 100-mer oligonucleotides in IP-RPLC. Therefore, the experimentally obtained model parameters (log *k*_0_, *S*) were expressed as a function of molecular weight (MW) for the set of poly-deoxythymidine oligonucleotides (Figure 1).

A linear relationship was observed. These results are consistent with the stoichiometric displacement theory (SDT) in which solvent and solute molecules compete for adsorption sites on the adsorbent surface.^30^ According to the SDT, retention in RPLC is described as a function of the number of solvent molecules (*Z*) required to displace the solute from the surface, where *Z* is directly proportional to the hydrophobic contact area between the solute and the stationary phase. When applied to IP-RPLC of oligonucleotides, *Z* is proportional to the number of hydrophobic TEA molecules involved in the ion-pairing process and thus to the length and MW of the homo-oligonucleotides. The chromatographic retention and associated log *k*_0_ values can then be expressed as a linear function of MW for a homologous series of oligonucleotides.

On the other hand, the sensitivity of retention to mobile phase composition (*S*) is known to be a solute-dependent parameter for given experimental conditions. Figure 1B illustrates the linearity between *S* values and MW of poly-dT oligonucleotides with a high correlation *R*^2^ > 0.99 for each mobile phase composition. There is no consensus in the literature on the relationship between *S* and MW for many chromatographic modes. In RPLC, the *S* values for small molecules are often approximated by the equation *S* ≈ 0.25 MW^0.5^,^31^ while a recent study reported different degrees of correlation between solute size and *S* based on a large set of small molecules and proteins.^21^ Together, these results confirm the dependence of *S* on experimental conditions and that one must be careful when approximating *S* values based on an empirical formula obtained with different mobile phase conditions.

An average decrease in *S* values by 40–60%, in combination with an increase in retention, was also observed when the amount of HFIP in the mobile phase was increased from 100 to 400 mM. The increased retention might be explained by the reduced mobile phase pH (measured pH values of 8.2–7.8 for 100–400 mM HFIP mobile phases, well below 9, p*K*_a_ of HFIP).^32^ This effect promotes ion-pair formation and thus increases oligonucleotide retention. According to this hypothesis, the increased sensitivity to the solvent composition for low-HFIP mobile phases could indicate a potential change in the partial molar volume of the oligonucleotides. This might result in a change in the energies of the adsorption–desorption interactions that would explain the increased *S* values.

### Illustration of the On–Off Mechanism of Oligonucleotides

In general, IP-RPLC acts as a mixed-mode chromatographic mode combining hydrophobic and electrostatic (ionic) retention mechanisms. However, if the ion-pairing reagent concentration is sufficiently high, elution is mainly driven by hydrophobic interactions occurring between the stationary phase ligands and the hydrophobic part (*i.e.*, alkyl chain) of the ion-pairing reagent molecules.

**Figure 2:**
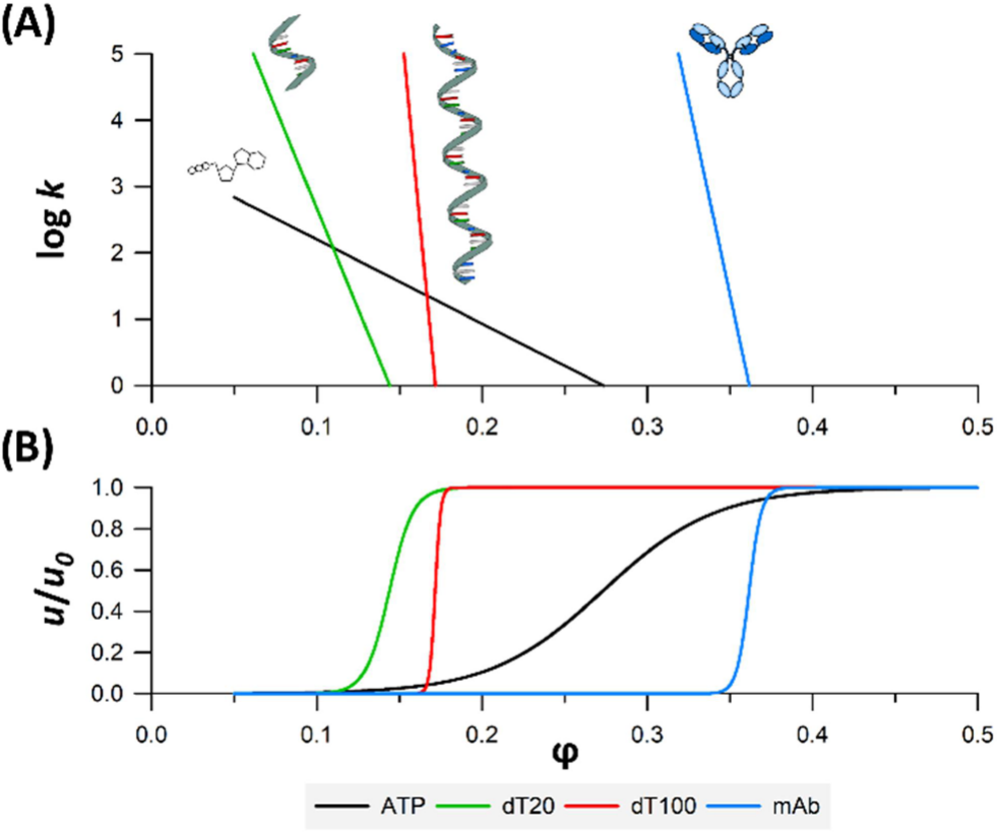
Logarithmic solute retention factor (A) and relative migration velocity (*u*/*u*_0_) (B) as a function of the volume fraction of the organic mobile phase φ for ATP (black, MW = 0.5 kDa; *S* = 13), dT20 (green, MW = 6 kDa; *S* = 60), dT100 (red, MW = 30 kDa; *S* = 264), and an intact mAb (blue, MW = 150 kDa, *S* = ∼116).

Figure 2A shows plots of the log retention factor as a function of mobile phase composition (φ) for ATP, dT20, dT100, and a monoclonal antibody (mAb) as a reference. The figure was constructed on the basis of data obtained with the 100 mM HFIP mobile phase, resulting in the highest *S* values. It clearly illustrates that dT20 already shows an on–off behavior (very steep curve, *S* = 60). For the dT100, we observed a 2.5 times higher *S* value (*S* = 264) compared with an intact mAb (*S* typically ranges between 100 and 150). The higher *S* values observed with ONs are probably due to the differences in molecular shape and specific surface available for interaction with the stationary phase (helical straight shape *versus* Y-shape).

Figure 2B shows the relative migration velocity as a function of mobile phase composition. Such a plot illustrates the transition between fully adsorbed (“on”) and fully released (“off”) states. The value of *S* = 264 (and the log *k*_0_ = 45.2) means that up to a mobile phase composition of φ = 0.164, the migration velocity of the dT100 is practically zero (bound at the column inlet, its velocity is less than 1% of the interstitial mobile phase velocity), whereas from φ = 0.179, it moves at a velocity greater than 99% of the mobile phase velocity, indicating that the solute is fully desorbed from the column. The transition range between the adsorbed and desorbed states therefore corresponds to a Δφ range of only 1.5% (0.179–0.164). The transition range for the dT20 is somewhat wider, namely, 6.5% of Δφ. This transition typically occurs between 3 and 4% Δφ for an intact mAb in RPLC mode. For the small ATP molecule, we observed a transition range of 31.4% Δφ (from φ = 0.116–0.430).

**Figure 3:**
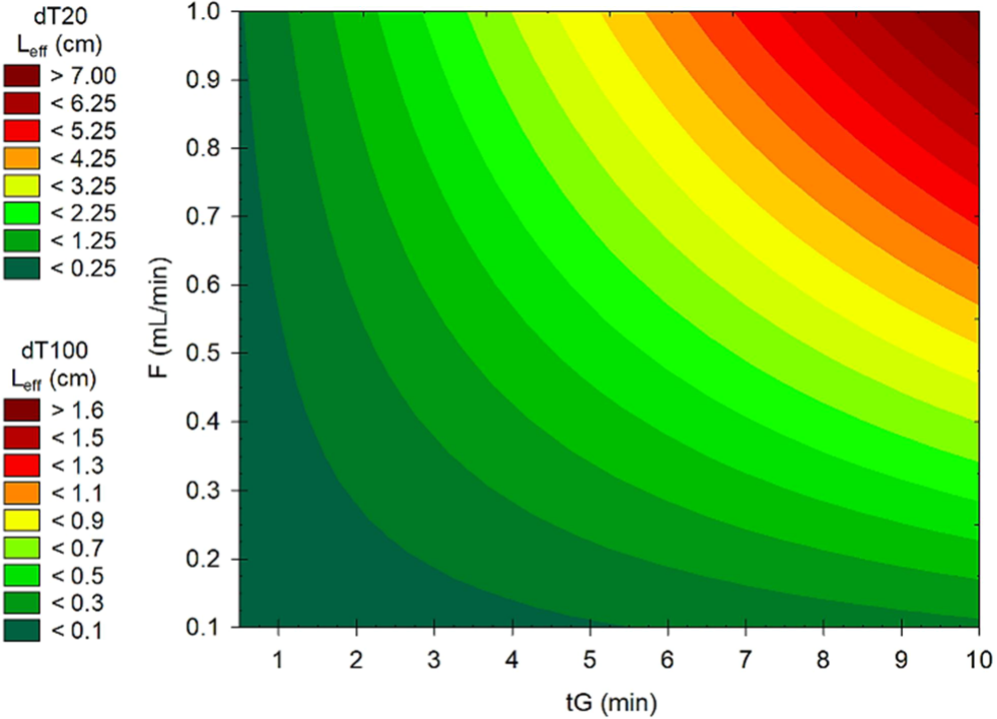
Color-coded surface plot of the column length (*L*_eff_) required to retain dT20 and dT100 with *k*_e_ = 0.5, as a function of gradient time and flow rate (*d*_c_ = 2.1 mm ID, ε = 0.68, and Δ*φ* = 0.2).

Figure 3 shows a plot of the column length (*L*_eff_) required to effectively retain dT20 and dT100 (at least at *k*_e_ = 0.5, which is a common practice for small molecule separations) as a function of gradient time and flow rate.

The calculations considered a column with *d*_c_ = 2.1 mm ID, total porosity ε = 0.68, and a linear mobile phase gradient Δφ = 0.2 (which is a rational choice for IP-RPLC separation of ONs). Based on the plot, running fast gradients (*i.e.*, *t*_G_ ≤ 3 min) does not require a column longer than 5 mm for dT100 and 22 mm for dT20. Experiments with low flow rates require even shorter columns. When using very fast gradients such as *t*_G_ = 1 min, only the first ∼3 mm or ∼10 mm segment of the column retains the dT100 or dT20 molecules, respectively. A 10 min long gradient at a flow rate of 0.5 mL/min would require a column *L* ≤ 10 mm for large ONs and a column *L* ≤ 35 mm for small ONs.

Based on these observations, it is clear that ONs in IP-RPLC conditions follow an on–off elution behavior and that for most linear gradient separation conditions a column length between 5 and 35 mm is appropriate. Longer columns will not improve selectivity or separation efficiency.

### Impact of Instrumentation on Apparent Efficiency

The success of ultra-short columns lies in minimizing extra-column band broadening induced by instrumentation. Indeed, the effect of extra-column band spreading is much more critical when using small-volume columns.

In LC, the total (observed) peak variance (σ_tot_^2^) is often expressed as the sum of the column variance (σ_col_^2^) and the extra-column variance (σ_ext_^2^), assuming normal distribution and independent sources of band broadening
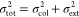
6

The volumetric peak variance due to dispersion occurring along a column can be expressed as
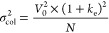
7where *N* is the column plate number (intrinsic efficiency), *V*_0_ is the column dead volume, and *k*_e_ is the retention factor of the solute at elution.

On the other hand, the extra-column variance depends on the instrument characteristics and can be considered as the sum of volume-based contributions, including injector (σ_inj_^2^), connection tubing (σ_tube_^2^), and UV cell (σ_cell_^2^), and time-based effects, such as the detector time constant (σ_tc_^2^),^33,34^ according to the following general equation
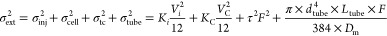
8

in which *K*_i_ and *K*_c_ are constants linked to the injection mode and the detector cell geometry, respectively. Apparent peak variance also depends on the injected volume (*V*_i_), flow-cell volume (*V*_c_), detector time constant (τ), flow rate (*F*), tubing diameter (*d*_tube_), length (*L*_tube_), and diffusion coefficient (*D*_m_).

As a rule of thumb, the overall extra-column variance should be no more than 10% of the column variance to maintain a reasonable loss of efficiency. However, the column dead volume and associated peak variance (eq 7) decrease in direct proportion to column length, so the effect of instrumentation on a very short column (*e.g.*, 5 mm long) can be critical. It is therefore of utmost importance to maintain a limited injection volume, shorten tubing, use low UV cell volume, and use appropriate UV time constant setting.

**Figure 4:**
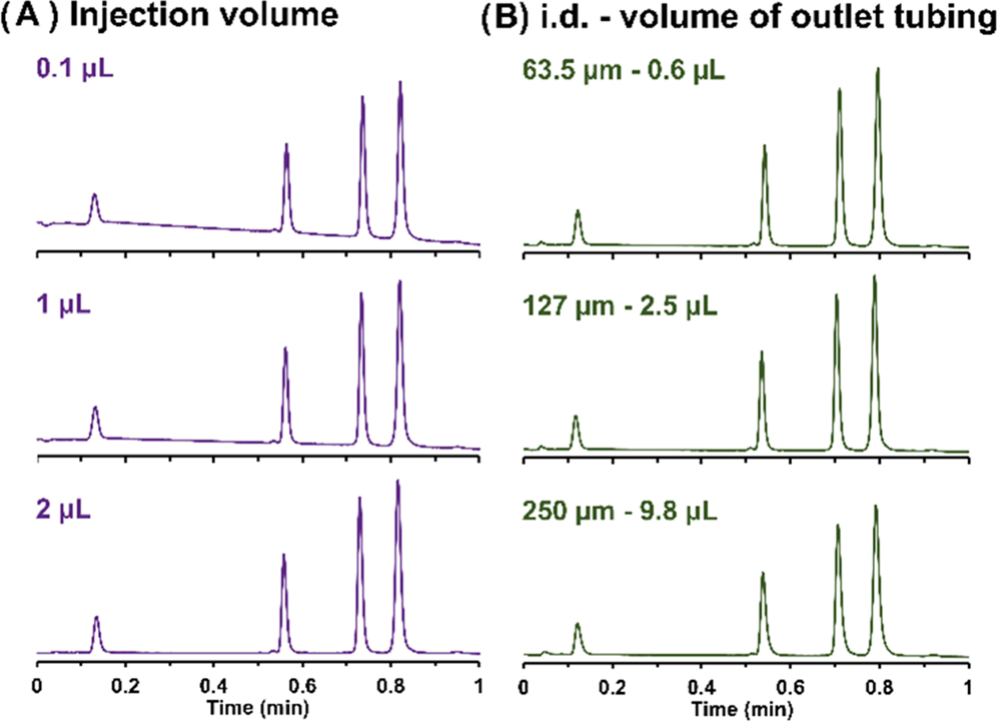
Comparative chromatograms obtained on a column of 5 mm length by injecting 0.1, 1, and 2 μL of a sample (A) and by using 63.5, 127, and 250 μm I.D. column outlet tubing of 20 cm long (B). Conditions: sample, 4 dT (dT10, dT20, dT30, dT40); gradient, 18–38%B in 1 min; flow rate, 1 mL/min.

First, the effect of injection volume on apparent efficiency was assessed, and corresponding chromatograms obtained with a mixture of four model ONs (dT10, dT20, dT30, and dT40) are shown in Figure 4A. All of the experiments were performed on a 5 mm × 2.1 mm I.D. column, with a dead volume of 12.7 μL. Thus, an injected volume of 1 μL already represents 10% of the column volume, which is clearly significant (not only for extra band broadening but also for column overload). Indeed, it is well known that the injection volume in LC should usually be ≈1–5% of the column volume to avoid excessive band broadening and peak distortion due to column overload.^35^

As shown in Figure 4A, the experimental peak capacities were equal to 33, 34, and 35 for injection volumes of 2, 1, and 0.1 μL, respectively. Thus, the impact of the injection volume on apparent efficiency was found to be negligible under the conditions tested. This behavior can probably be explained by the fact that all of the experiments were performed in gradient elution mode and relatively low injection volumes require very short loading times at the selected flow rate (fast, instant injection). In such conditions, bandwidths are refocused at the column inlet due to the very high retention factor experienced in the initial mobile phase composition (thanks to the on–off mechanism). Consequently, the precolumn and column-inlet band broadening is compensated for by this refocusing effect.^36^ In addition, in the present work, there is a difference between the sample diluent (purely aqueous) and the mobile phase (around 10–20% organic solvent in water + salts). The small difference in composition between the sample diluent and the mobile phase produces a strong difference in eluent strength for ONs, which are subjected to an on−off retention mechanism (see the section Illustration of the On–Off Mechanism of Oligonucleotides). So, it is an additional contribution to eliminate precolumn broadening.^37^ In the end, a relatively large volume of 2 μL can be injected on the 5 mm column length to maximize sensitivity, without any additional band broadening.

Besides the injection volume, we have also evaluated the effect of outlet tubing on peak capacity using the same mixture of model ONs and the 5 mm × 2.1 mm I.D. column. The inlet tubing located between the injector outlet and the column inlet cannot be easily modified on our instrument as it contains an active preheater, which is mandatory when working at elevated temperatures (beyond 40 °C). In addition, this tubing should have a very limited impact on band broadening, due to the strong focusing effect occurring at the column inlet (see the above discussion). Therefore, only the outlet tubing (between the column outlet and the UV detector) was modified. The shortest possible length was systematically used (20 cm), and three different internal diameters were evaluated (i.e., 63.5, 127, and 250 μm). The corresponding tube volumes were equal to 0.6, 2.5, and 9.8 μL. As shown in eq 8, a longer and/or wider flow path typically results in more dispersion. If residence time in the tube and/or the tube length are long enough, the dispersion should increase as the inner diameter of the connecting tubing increases (proportional to *d*_tube_^4^). Thus, broader peaks are expected with larger tubing as a result of the parabolic flow profile (Poiseuille, laminar regime) that is established in an open and straight tubing.^38^ In addition, eq 8 also suggests that low diffusion coefficients may be responsible for an increase in band broadening. Since ONs, due to their large sizes (from 3 to 30 kDa), possess relatively low diffusivity, the negative impact of the tube diameter on apparent efficiency may be even greater. However, in eq 8, the dispersion contribution of the tube is given by the Taylor–Aris equation (very often used in LC),^39^ which is only adequate if certain requirements (so-called Taylor conditions) are fulfilled (e.g., long enough tube and residence time, when operating at low flow rates). In our case, the tube employed at the column outlet was short and straight, while the flow rate was relatively high (1 mL/min), thus resulting in very short residence times (between 0.04 and 0.5 s, depending on tube diameter), and thus Taylor conditions were not fulfilled.

In the end, Figure 4B shows that the experimental chromatograms were not strongly affected by the dimensions of the outlet tubing, despite the contribution of tube diameter described in eq 8, because of the high flow rate applied and the very low diffusion coefficient of the solutes. Also note that in the case of the on–off elution mechanism, the released ON peaks leave the column end with a very high velocity (they are not retained), which results in an apparent band compression in the time domain (i.e., a peak possessing a given axial spatial width leaves the column in a very short time), thus resulting in apparent very sharp peaks. This effect probably also contributes to our observation that post-column tubing had a very minor impact on apparent efficiency. The observation would probably be different with a small molecule that leaves the column with a significantly lower speed or when working at a low flow rate. In the end, the peak capacity values vary between 34 for the 63.5 and 127 μm I.D. tube and 30 for the 250 μm tubing, at a flow rate of 1 mL/min, so only a minor loss in performance was observed with the 250 μm tubing, despite the fact that it has a volume comparable to the 5 mm column volume (*i.e.*, 12.7 μL). Finally, it seems that there is no need to use very narrow tubing when analyzing ONs with ultra-short columns at a high flow rate. A short outlet tubing with an inner diameter of 127 μm can be perfectly adapted and avoids excessive external pressure (the pressure generated by an open tube is inversely proportional to the diameter of the tube with a power of 4, according to the Hagen–Poiseuille equation).

### Impact of Column Length on Apparent Efficiency

**Figure 5:**
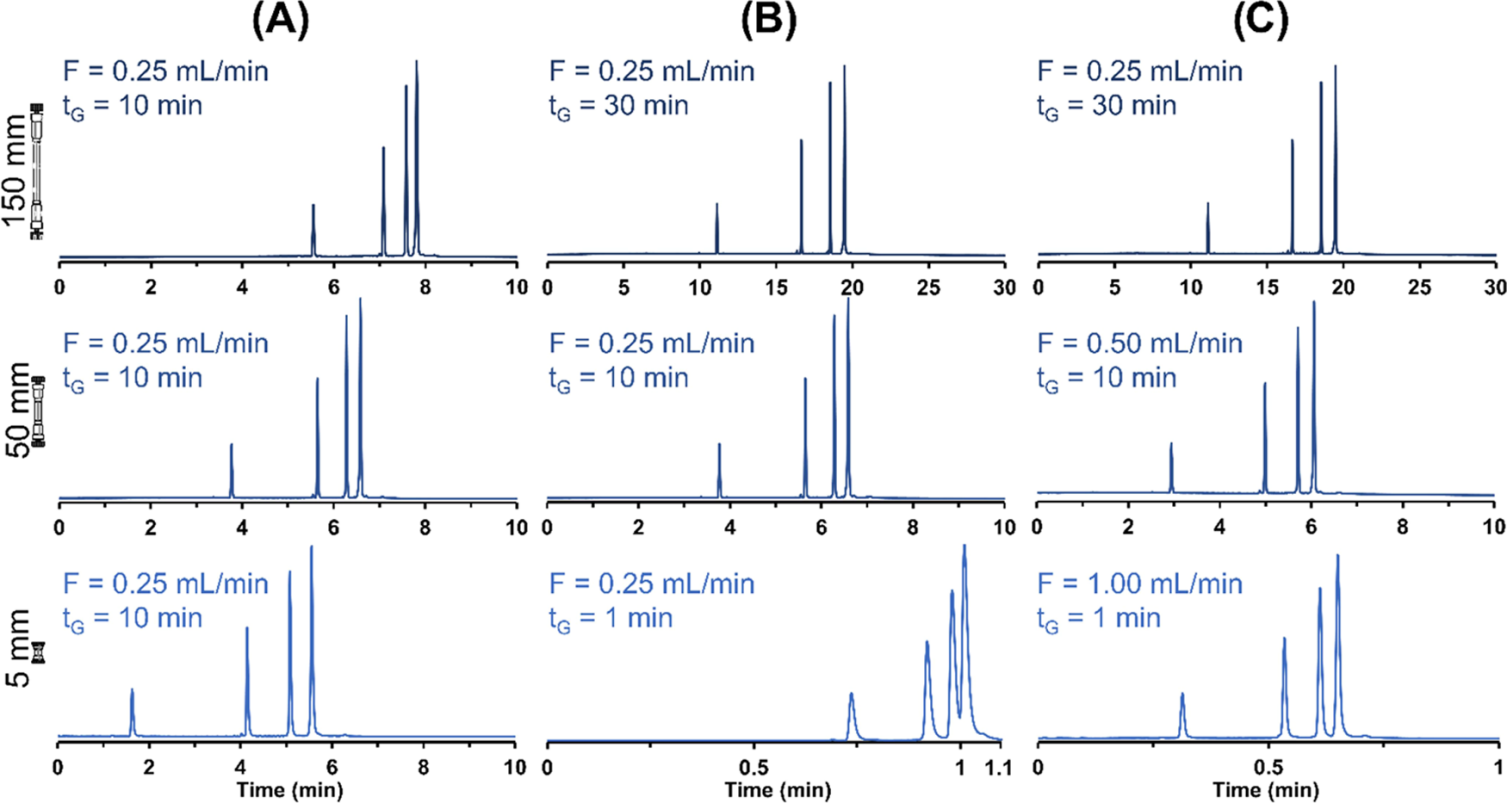
Comparative chromatograms obtained on columns of 150, 50, and 5 mm length at *F* = 0.25 mL/min and *t*_G_ = 10 min (A), at *F* = 0.25 mL/min and *t*_G_ = 30, 10, and 1 min (B), and at *F* = 0.25, 0.50, and 1 mL/min and *t*_G_ = 30, 10, and 1 min (C). Conditions: sample, 4 dT (dT10, dT20, dT30, dT40); gradient, 10–50%B.

To evaluate the interest in ultra-short columns for the analysis of ONs, three different series of experiments were performed. In the first series (Figure 5A), the flow rate (0.25 mL/min) and gradient time (10 min) were kept constant and only the column length was varied (150, 50, and 5 mm). The corresponding minimum peak capacity (*P*_min_) and peak capacity (*P*) values are reported in Table S5. Under these conditions, the highest peak capacity (calculated from the average peak widths of the four peaks and the difference in retention times between the first and last peaks) was obtained with the 5 and 50 mm columns (*P* values of 54 and 56, respectively), while the value obtained with the 150 mm column was lower (*P* = 38). This is due to the fact that the peak capacity is inversely related to the column dead time (column dead time is reduced by a factor of 30 between 150 and 5 mm column length), so the intrinsic gradient steepness is very different when changing the column length.^40^ Therefore, when fast gradients are performed, the longest column is not necessarily the most efficient. An additional advantage observed with the 5 mm long column is the favored distribution of the peaks in a wider retention window (higher selectivity). To highlight this point, the minimum peak capacity, *P*_min_, was calculated from the average peak widths of dT20 and dT30 and the difference in retention time between this pair.^25^ It appears that *P*_min_ was equal to 7.4, 6.9, and 4.8 on the 5, 50, and 150 mm columns, respectively. These results show that ultra-short columns of only 5 mm may be interesting to maximize selectivity and overall resolution in gradient mode while maintaining reasonable analysis times (only 10 min). Similar *P*_min_ values can certainly be obtained on the 150 mm long column but with much longer analysis times. The chromatograms reported in Figure 5A illustrate the on−off retention mechanism of ONs under IP-RPLC conditions.

In the second series of experiments, the flow rate was kept constant and the gradient time was scaled in direct proportion to the column length. Gradient times of 30, 10, and 1 min were used with the 150, 50, and 5 mm long columns, respectively. The corresponding chromatograms are shown in Figure 5B. In this case, the average peak capacities were strongly modified depending on the analytical conditions and were equal to 85, 56, and 14 on the 150, 50, and 5 mm columns, respectively. P_min_ was also strongly modified, decreasing from 10.7 on the longest column to only 2.3 on the shortest one. This behavior is expected since an extension of the gradient time is always beneficial for the peak capacity, regardless of the column length. The overall performance attained with the 5 mm column was therefore particularly low at a flow rate of only 0.25 mL/min, with no baseline resolution between dT30 and dT40. Under the conditions used in Figure 5B, the pressure observed on the 5 mm column length was greatly reduced (by a factor of 30) compared with the longest column. This means that the potential of the 5 mm long column is not fully exploited, according to the kinetic plot methodology.^41,42^

For this reason, a third set of experiments was performed, by increasing the flow rate as the column length decreased. Figure 5C shows the corresponding chromatograms obtained under these conditions. The peak capacity obtained on the 50 mm column does not vary significantly with flow rate (*P* = 56 and 57 at 0.25 and 0.5 mL/min, respectively) but was almost doubled on the 5 mm long column (*P* = 14 and 26 at 0.25 and 1 mL/min, respectively).

Under the selected conditions, the dead time of the 5 mm column at 1 mL/min was extremely low and equal to 0.64 s, so it was quite easy to reach analysis times below 1 min. As illustrated in Figure 5B,C, to take full advantage of the ultra-short columns, it is essential to increase the flow rate when combining short columns and fast gradients.

### High-Throughput Separations on a 5 mm Column

A final set of experiments was performed with three different ON samples, including a mixture of small ONs (*i.e.*, dT10, dT20, dT30, and dT40), large ONs (i.e., dT40, dT60, dT80, and dT100), and a mixture of 20-mer ONs with a different number of phosphorothioate (PS) moieties (i.e., dT20, dT20–9PS, dT20–6PS, and dT20–19PS). These three samples were analyzed under two different conditions: (i) reference conditions which consisted of using a 5 mm long column, at a flow rate of 1 mL/min, a temperature of 60 °C, and a gradient time of 1 min and (ii) extreme conditions which consisted of using a 5 mm long column, at a flow rate of 1.75 mL/min, a temperature of 90 °C, and a gradient time of 30 s. Corresponding peak capacity values are reported in Table S5.

**Figure 6:**
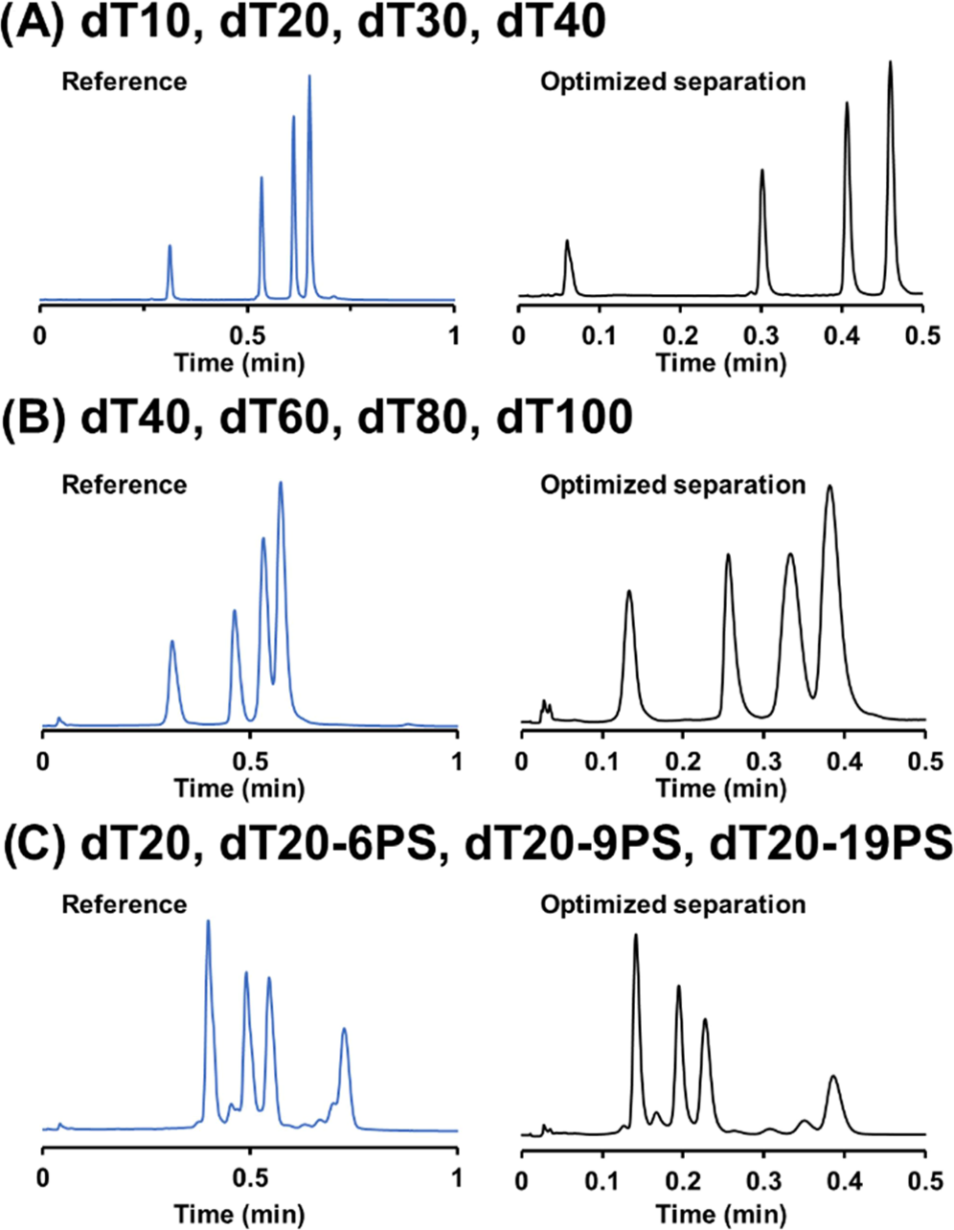
Reference (blue trace) and optimized (black trace) separations on a 5 mm column. Conditions (reference *vs* optimized): 60 °C *vs* 90 °C, 1 mL/min *vs* 1.75 mL/min, *t*_G_ = 1 min *vs* 30 s. Gradient windows are reported in the Experimental Section. Samples: 4 dT—dT10, dT20, dT30, dT40 (A), dT40–100—dT40, dT60, dT80, dT100 (B), and 4 PS—dT20, dT20–6PS, dT20-9-PS, dT20–19PS (C).

Under the reference conditions, the chromatograms corresponding to the three mixtures of ONs are shown in Figure 6 (left panels). The peak capacity was higher for the mixture of small ONs (*P* = 26), while it drops to only 8 for the mixture of large ONs and 10 for the mixture of PS ONs. The same observations were found for *P*_min_ with values of 2.1, 2.4, and 3.8 for large ONs, PS ONs, and small ONs, respectively.

The lower performance observed for the large ONs was probably attributed to (i) the pore size of the column (130 Å), which was probably too small for the analysis of large ONs,^43^ and (ii) the lower *D*_m_ of large ONs, which means that the selected flow rate was probably far from the optimal one. On the other hand, the low peak capacity observed with PS ONs has been attributed to the presence of numerous diastereoisomers in the sample that are partially resolved under IP-RPLC conditions, leading to peak broadening.^25,44^ Peak widths were measured for dT20 and the phosphorothioated ONs. For dT20–6PS, dT20–9PS, and dT20–19PS, peak broadening was increased by only 10, 12, and 30%, respectively, in comparison with the reference dT20, which is reasonable for phosphorothioated ONs. These narrow peaks were due to the elevated mobile phase temperature employed in this work (60 °C), limiting the selectivity for diastereomers of phosphorothioated ONs. Interestingly, the separation of the 4 dT20 with differing numbers of phosphorothioates offers a relatively good selectivity, which may be attributed to a change in hydrophobicity between the phosphodiester and phosphorothioate moiety.

To further improve the overall kinetic performance and simultaneously reduce the analysis time, some extreme conditions were applied, and the corresponding chromatograms are shown in Figure 6 (right panels). Analysis times were reduced by a factor of about 2, and the peak capacity increased by an average of approximately 20%. The change in overall resolution (expressed as *P*_min_) was negligible for the large ONs (*P*_min_ changed from 2.1 to 2.2) and PS ONs (*P*_min_ = 2.4 in both cases), while the improvement was noticeable for the small ONs (*P*_min_ varied from 3.8 to 5.2). In addition, due to the increase of mobile phase temperature from 60 to 90 °C and the associated decrease in viscosity, the overall pressure did not change at 1.75 mL/min and 90 °C compared with 1 mL/min and 60 °C. This indicated that increased temperature and high flow rates are beneficial when using ultra-short columns to maximize kinetic performance and resolving power. In addition, it is important to mention that binary pumping systems have to be preferentially used to achieve good repeatability under such extreme conditions.

## Conclusions

The goal of this proof-of-concept work was to evaluate if ultra-short columns can be used in IP-RPLC for the analysis of model oligonucleotides in gradient elution mode.

First, the validity of the LSS model was evaluated, and the accuracy of retention time predictions was assessed. It was found that ONs in IP-RPLC conditions follow the on–off elution behavior and that for most linear gradient separation conditions an effective column length between 5 and 35 mm was sufficient to obtain the best selectivity and separation efficiency. Therefore, the applicability of ultra-short columns was explored to speed up ON separations. In this regard, the extra-column band broadening induced by the instrumentation was carefully evaluated. Interestingly, the impact of injection volume and post-column connection tubing on peak capacity were found to be negligible. Next, the impact of column length on apparent efficiency was also monitored. First, column length was varied (150, 50, and 5 mm), while flow rate and gradient time were kept constant. Then, various gradient times and flow rates were tested on the three column lengths. In all cases, the on–off retention mechanism of ONs was confirmed. In addition, 5 mm column proved to be an advantageous choice to (i) increase the overall resolution achievable in gradient mode and (ii) perform ultra-fast separations with reasonable kinetic performance.

Optimized ultra-fast separations were therefore developed on the 5 mm column by using a flow rate of 1 mL/min and a gradient time of 1 min. Three model oligonucleotide mixtures including small, large, and PS-modified ONs were analyzed, and a remarkable resolution was especially obtained for the separation of the small ONs mixture. The separations were subsequently pushed to their limits at a higher flow rate (1.75 mL/min) and temperature (90 °C against the previous 60 °C), enabling baseline separation of all of the mixtures in as little as 30 s.

For the first time, a comprehensive study on the demonstration of the applicability of the LSS model to model oligonucleotides’ IP-RPLC separations in gradient elution mode was performed. The understanding of the ONs retention behavior enabled the evaluation of ultra-short columns and the selection of a 5 mm column to boost the ONs baseline separations in less than 1 min. These findings represent the first proof of concept for achieving ONs high-throughput analysis and pave the way for further investigation using more complex therapeutic oligonucleotides.

Interestingly, there are two possible reasons for using ultra-short columns for ONs: (i) achieving ultra-fast separation (1 min or less), while maintaining a reasonable efficiency, provided that the flow rate is sufficiently high and (ii) increasing the overall resolution achievable in gradient mode (corresponding to *P*_min_) within a reasonable analysis time (few minutes). The second aspect has not yet been explored but will be investigated in an upcoming work.

## Supporting Information Available

Experimental conditions to derive LSS parameters; log *k*_0_ and *S* values corresponding to Figure 1; data to verify the adequacy of the LSS model; experimental conditions to study the impact of instrumentation (PDF)

## Notes

S.F. and M.L. are employees of Waters (Milford, MA), who have produced the columns employed in this work.
