# Supplementary material for: High-Throughput Chromatographic Separation of Oligonucleotides: A Proof of Concept Using Ultra-Short Columns
Source: Anal Chem. 2023 Jun 29;95(27):10448–56. doi: 10.1021/acs.analchem.3c01934 (PMC10339280; doi:10.1021/acs.analchem.3c01934)
Supplement: Supplementary file 1 — ac3c01934_si_001.pdf [file ac3c01934_si_001.pdf]

Supplementary Data

High-throughput chromatographic separation of oligonucleotides: a proof of concept using ultra-short columns

Honorine LARDEUX [a, b], Szabolcs FEKETE [c], Matthew LAUBER [d], Valentina D'ATRI [a, b], Davy GUILLARME [a, b, *]

[a] *Institute of Pharmaceutical Sciences of Western Switzerland (ISPSO), University of Geneva, Geneva, 1211, Switzerland.*

[b] *School of Pharmaceutical Sciences, University of Geneva, Geneva, 1211, Switzerland.*

[c] *Waters Corporation, located in CMU-Rue Michel Servet 1, Geneva, 1211, Switzerland.*

[d] *Waters Corporation, Milford, MA 01757, USA.*

* Corresponding author:

Davy GUILLARME

E-mail address: davy.guillarme@unige.ch (D. Guillarme).

TABLE OF CONTENTS

**Table S1**	Experimental conditions to derive LSS parameters	S2
**Table S2**	Log k_0 and S values corresponding to Figure 1 and based on the retention data generated using experimental conditions detailed in Table S1..........	S3
**Table S3**	Predicted retention times, experimentally observed retention times and corresponding errors (%) to verify the adequacy of the LSS model............	S4
**Table S4**	Experimental conditions to study the impact of instrumentation..............	S6
**Table S5**	Average minimum peak capacity (Pmin) and peak capacity (P) values obtained for the various experiments performed using columns of different lengths..	S7

Table S1. Experimental conditions to derive LSS parameters.

	Adenosine triphosphate (ATP)	dT10-40 (dT10, dT15, dT20, dT25, dT30, dT35, dT40)	dT40-100 (dT40, dT60, dT80, dT100)
Diluent (concentration)	water (5 μM)	water (5 μM)	water (5 μM)
Injection volume	1 μL	1 μL	1 μL
Column	Acquity™ Premier™ Oligonucleotide BEH C18 2.1 x 50 mm	Acquity™ Premier™ Oligonucleotide BEH C18 2.1 x 50 mm	Acquity™ Premier™ Oligonucleotide BEH C18 2.1 x 50 mm
Mobile phases	A = 100 mM HAA* in H_2O, pH 7<br>B = 50%A 50% ACN<br>**hexylammonium acetate used as additive to sufficiently retain ATP*	A = 14 mM TEA, 160 mM* HFIP in H_2O<br>B = 50%A 50% MeOH<br>**100 and 400 mM HFIP were also tested, with the same gradient*	A = 14 mM TEA, 160 mM* HFIP in H_2O<br>B = 50%A 50% MeOH<br>**100 and 400 mM HFIP were also tested, with the same gradient*
Gradient	10-50%B (5-25%ACN)<br>t_G = 30, 10 min	10-50%B (5-25%MeOH)<br>t_G = 30, 10 min	30-70%B (15-35%MeOH)**<br>t_G = 30, 10 min<br>***except for MP containing 100 mM HFIP: 20-60%B (10-30%MeOH)*
Flow rate	0.5 mL/min	0.5 mL/min	0.5 mL/min
Col. Temperature	60 °C	60 °C	60 °C

Table S2. Log k_0 and S values corresponding to Figure 1 and based on the retention data generated using experimental conditions detailed in Table S1.

Solute	MW (Da)	Mobile phase	Log k_0	S
ATP	507	100 mM HAA	3.47	12.7
dT10	2980	100 mM HFIP	4.35	37.7
		160 mM HFIP	3.79	29.6
		400 mM HFIP	4.18	23.7
dT15	4501	100 mM HFIP	6.60	49.4
		160 mM HFIP	6.05	40.2
		400 mM HFIP	6.68	33.2
dT20	6022	100 mM HFIP	8.71	60.5
		160 mM HFIP	8.31	51.3
		400 mM HFIP	9.00	42.0
dT25	7543	100 mM HFIP	10.84	72.0
		160 mM HFIP	10.64	62.9
		400 mM HFIP	11.15	50.0
dT30	9064	100 mM HFIP	13.33	86.1
		160 mM HFIP	12.15	69.6
		400 mM HFIP	13.24	57.8
dT35	10585	100 mM HFIP	15.01	94.7
		160 mM HFIP	15.32	86.4
		400 mM HFIP	15.21	65.2
dT40	12106	100 mM HFIP	17.50	109.2
		160 mM HFIP	17.31	90.7
		400 mM HFIP	17.66	74.7
dT60	18190	100 mM HFIP	26.10	156.8
		160 mM HFIP	26.65	134.9
		400 mM HFIP	26.24	107.5
dT80	24274	100 mM HFIP	34.06	200.8
		160 mM HFIP	36.63	182.3
		400 mM HFIP	35.17	141.9
dT100	30358	100 mM HFIP	45.22	263.6
		160 mM HFIP	48.17	237.4
		400 mM HFIP	44.08	176.1

Table S3. Predicted retention times ($t_{r,pred}$), experimentally observed retention times ($t_{r,exp}$) and corresponding errors (%) to verify the adequacy of the LSS model.

Mixture	Solute	Gradient time (min)	$t_{r,pred}$ (min)	$t_{r,exp}$ (min)	Error (%)
ATP	ATP	60	28.41	28.176	0.80%
dT15-40	dT10	60	10.94	10.691	2.30%
	dT15	60	21.79	21.899	0.50%
	dT20	60	27.75	27.882	0.50%
	dT25	60	31.44	31.516	0.20%
	dT30	60	33.92	33.964	0.10%
	dT35	60	35.69	35.707	0.00%
	dT40	60	37.04	37.035	0.00%
dT40-100	dT40	60	9.98	10.061	0.80%
	dT60	60	13.22	13.269	0.40%
	dT80	60	14.82	14.857	0.20%
	dT100	60	15.78	15.801	0.10%
ATP	ATP	20	13.36	13.23	1.00%
dT15-40	dT10	20	5.44	5.381	1.10%
	dT15	20	8.72	8.722	0.00%
	dT20	20	10.46	10.447	0.10%
	dT25	20	11.51	11.501	0.10%
	dT30	20	12.22	12.212	0.10%
	dT35	20	12.73	12.718	0.10%
	dT40	20	13.11	13.10	0.10%
dT40-100	dT40	20	4.13	4.121	0.20%
	dT60	20	5.04	5.031	0.20%
	dT80	20	5.48	5.475	0.10%
	dT100	20	5.74	5.738	0.00%
ATP	ATP	15	10.85	10.768	0.80%
dT15-40	dT10	15	4.49	4.453	0.80%
	dT15	15	6.88	6.877	0.00%
	dT20	15	8.13	8.121	0.10%
	dT25	15	8.89	8.876	0.20%
	dT30	15	9.40	9.383	0.20%
	dT35	15	9.76	9.745	0.20%
	dT40	15	10.03	10.019	0.10%
dT40-100	dT40	15	3.30	3.295	0.20%
	dT60	15	3.95	3.945	0.10%

	dT80	15	4.26	4.26	0.00%
	dT100	15	4.45	4.444	0.10%
ATP	ATP	5	4.82	4.869	1.00%
dT15-40	dT10	5	2.17	2.186	0.70%
	dT15	5	2.87	2.878	0.30%
	dT20	5	3.22	3.229	0.30%
	dT25	5	3.43	3.439	0.30%
	dT30	5	3.57	3.578	0.20%
	dT35	5	3.67	3.676	0.20%
	dT40	5	3.74	3.749	0.20%
dT40-100	dT40	5	1.51	1.519	0.60%
	dT60	5	1.68	1.691	0.70%
	dT80	5	1.76	1.770	0.60%
	dT100	5	1.81	1.814	0.20%

Table S4. Experimental conditions to study the impact of instrumentation.

	4dT (dT10, dT20, dT30, dT40)
Diluent (concentration)	water (5-µM)
Injection volume	0.1, 1 and 2 µL were tested (Figure 3A), otherwise 1 µL
Column	Acquity™ UPLC™ BEH C18 1.7 µm, 130 Å VanGuard 5 × 2.1 mm pre-column
Mobile phases	A = 14 mM TEA, 100 mM HFIP in H_2O<br>B = 50%A 50% MeOH
Gradient	18-38%B (9-19%MeOH)<br>t_G = 1 min
Flow rate	1 mL/min
Col. Temperature	60 °C
20-cm column outlet tubing	i.d. of 63.5, 127 and 250 µm were tested (i.e 0.6, 2.5 and 9.8 µL tubing, Figure 3C), while 63.5 µm tubing was used for previous analyses.

Table S5. Average minimum peak capacity (P_{min}) and peak capacity (P) values obtained for the various experiments performed using columns of different lengths.

	P_{min}	P
Figure 5A - 150 mm	4.8	38
Figure 5A - 50 mm	6.9	56
Figure 5A - 5 mm	7.4	54
Figure 5B - 150 mm	10.7	85
Figure 5B - 50 mm	6.9	56
Figure 5B - 5 mm	2.3	14
Figure 5C - 150 mm	10.3	85
Figure 5C - 50 mm	7.2	57
Figure 5C - 5 mm	3.7	26
Figure 6A - Reference	3.8	26
Figure 6A - Optimized	5.2	31
Figure 6B - Reference	2.1	8
Figure 6B - Optimized	2.2	9
Figure 6C - Reference	2.4	10
Figure 6C - Optimized	2.4	13
